# Spatial oligonucleotide immuno-reprogramming model (SOIRM): a testable framework linking bone marrow resistance states and oligonucleotide therapeutics in multiple myeloma

**DOI:** 10.3389/fimmu.2026.1917518

**Published:** 2026-10-01

**Authors:** Ahmed M. Hjazi

**Affiliations:** 1 Department of Medical Laboratory, College of Applied Medical Sciences, Prince Sattam bin Abdulaziz University, Al-Kharj, Saudi Arabia; 2 Royal Diagnostics Medical Laboratory, Riyadh, Saudi Arabia

**Keywords:** bone marrow microenvironment, measurable residual disease, multiple myeloma, oligonucleotide therapeutics, single-cell sequencing, SOIRM, spatial transcriptomics, therapeutic resistance

## Abstract

This narrative review presents SOIRM as a conceptual and testable research framework rather than a systematic review, a report of new experimental data, or an established treatment model. It distinguishes findings supported by multiple myeloma studies from emerging evidence, the author’s interpretation, and hypotheses that require dedicated experimental and clinical validation. Therapeutic resistance and measurable residual disease (MRD) in multiple myeloma (MM) reflect tumor-intrinsic evolution and interactions between malignant plasma cells and the bone marrow microenvironment. Recent primary studies have identified inflammatory stromal populations, subclone-specific effects on the microenvironment, site-specific plasma-cell programs, and immune states associated with treatment response. These findings do not yet establish a clinically applicable spatially targeted intervention. The Spatial Oligonucleotide Immuno-Reprogramming Model (SOIRM) is therefore proposed as a falsifiable research framework, not as an established therapy.

## Graphical Abstract

**Figure:**
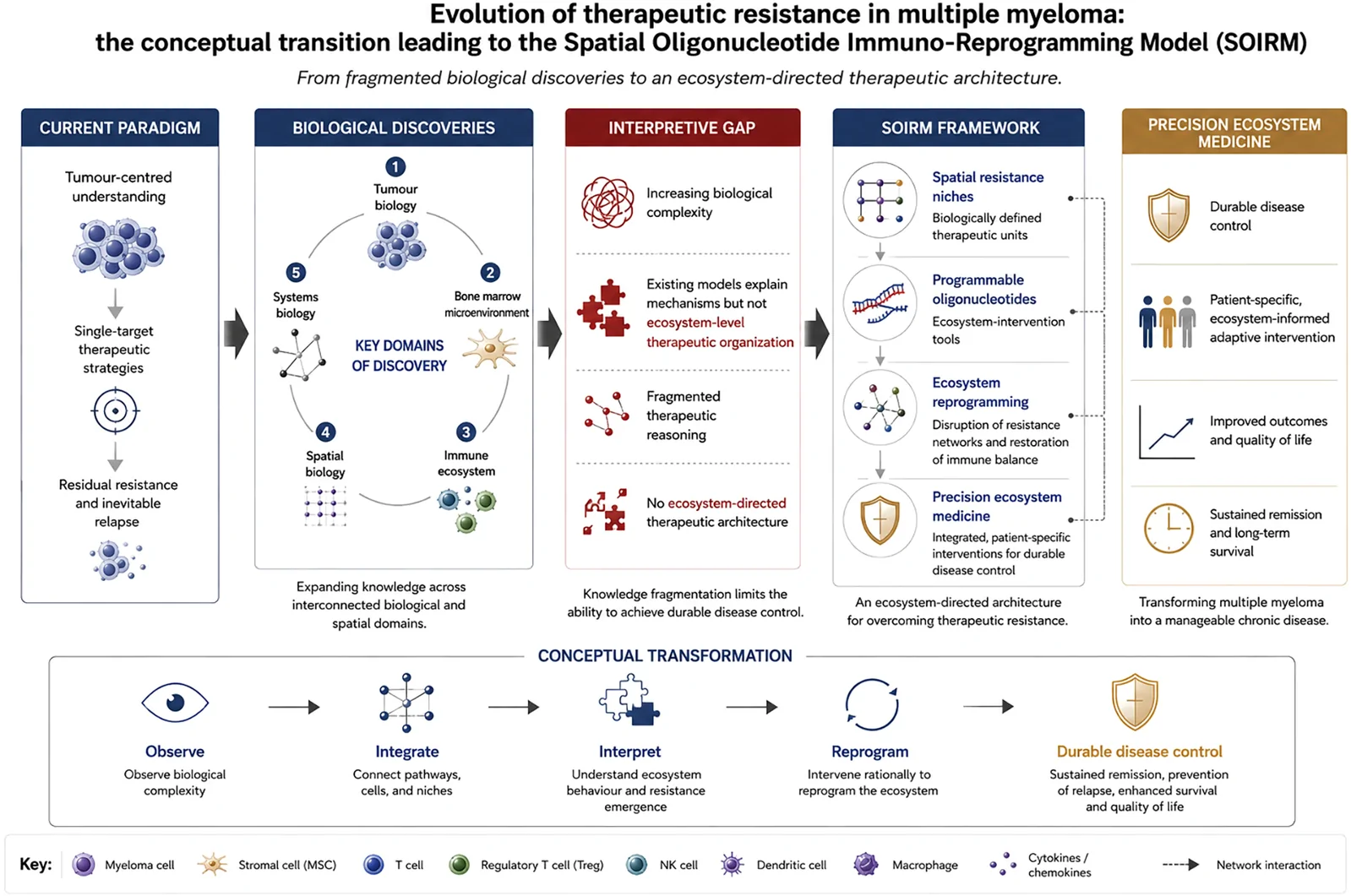
Development of the SOIRM research framework. The figure summarizes how evidence from tumor biology, the bone marrow microenvironment, immunology, spatial profiling, and oligonucleotide therapeutics informed the proposed SOIRM framework. The schematic integrates evidence-supported component mechanisms with hypotheses linking spatially defined resistance-associated states to candidate oligonucleotide interventions. These hypotheses require functional and clinical validation and should not be interpreted as established treatment pathways.

## 1 Introduction

Treatment of MM has been transformed by proteasome inhibitors, immunomodulatory drugs, anti-CD38 antibodies, BCMA-directed cellular therapies, and T-cell engagers; however, relapse remains common, and responses vary among patients with apparently similar disease. Tumor-intrinsic clonal evolution is a major explanation, but multi-region and longitudinal sampling also show that a single iliac crest aspirate may not represent disease distributed across focal lesions. In 24 patients followed for up to 14 years, sequencing of 140 spatially and longitudinally collected samples identified distinct relapse patterns, including geographically separated subclones and resistant populations that remained undetected for years (1). This finding supports spatially structured evolution but does not show that every relapse is driven by the microenvironment.

Primary studies provide additional evidence of interactions between myeloma cells and the bone marrow microenvironment. Stromal co-culture induced extensive changes in chromatin accessibility in MM cells and a migration- and cytokine-related program associated with dissemination and early resistance in patient cohorts (2). Single-cell analysis of refractory MM revealed that individual subclones can exhibit distinct microenvironmental changes and drug responses (3). Other studies identified inflammatory stromal populations in patient marrow (4) and an IL-1β-dependent neutrophil-stromal pathway associated with a BAFF-rich protumor microenvironment (5). These findings support reciprocal tumor-stromal and immune-stromal interactions; they do not establish a single composite score for the entire microenvironment.

Spatial and single-cell studies provide disease-specific examples. Paired profiling of focal lesions and marrow identified substantial transcriptional differences despite limited genomic divergence in some patients (6). Multi-region single-cell RNA and ATAC sequencing in 17 newly diagnosed patients identified a median of six tumor subclones per patient, lesion-specific subclones, variable expression of therapeutic targets, and regional differences in macrophage abundance (7). These observations support direct measurement of tissue organization when evaluating treatment resistance.

Oligonucleotide therapeutics permit sequence-specific modulation of selected targets, but direct evidence in MM remains limited. Formulated miR-34a inhibited MM growth in preclinical models (8), and a nuclease-resistant BCMA RNA aptamer was internalized by BCMA-positive myeloma cells and delivered miR-137 or anti-miR-222 cargo (9). By contrast, clinical studies of the BCL2 antisense oligonucleotide oblimersen did not establish a practice-changing therapeutic benefit (10, 11). These findings support sequence-specific target modulation and receptor-directed delivery, but not coordinated modification of the tumor and its microenvironment.

SOIRM is therefore presented as a research hypothesis linking spatially defined resistance-associated states to programmable molecular perturbation. Its value depends on generating testable predictions and providing information beyond tumor genotype, disease burden, and established microenvironmental biomarkers. The oligonucleotide platforms and proposed applications are summarized in **Table 1**.

**Table 1:** Oligonucleotide platforms and proposed applications in multiple myeloma.

Dominant biological function	Programmable oligonucleotide platform	Primary mechanistic action	Principal spatial resistance niche	Interpretation within SOIRM	Proposed measurable outcome	Evidence status and proposed scope
Suppression of stromal communication	Antisense oligonucleotides (ASOs)	RNase H-mediated transcript degradation or steric-blocking splice modulation	Cytokine-Survival Niche	Proposed to weaken selected stromal signaling pathways	Reduced stromal dependence and increased treatment sensitivity	Potentially broad; unvalidated
Disruption of resistance-supporting communication hubs	Small interfering RNAs (siRNAs)	Sequence-specific RNA interference–mediated gene silencing	Retention, Immune-Checkpoint, and MRD-Persistence Niches	Proposed to silence selected regulators of cell-cell or cell-matrix interactions	Loss of adaptive signalling networks and increased treatment susceptibility	Potentially broad; unvalidated
Modulation of multiple regulatory pathways	microRNA therapeutics	Coordinated modulation of multiple regulatory pathways	Immune-Suppression and Osteoimmune Niches	Hypothesized multi-pathway modulation; coordinated effects across malignant and nonmalignant compartments have not been demonstrated	Potential disruption of adaptive resistance; multicompartment benefit remains untested	Multicompartment effect hypothesized
Activation of antitumor immune responses	CpG oligonucleotides (CpG ODNs)	TLR9-mediated activation of innate and adaptive immunity	Immune-Suppression Niche	Proposed to enhance local antitumor immunity by reducing immune suppression	Enhanced immune surveillance and improved antitumour immune activity	Immune-cell effect proposed
Cell-selective oligonucleotide delivery	Aptamer-guided oligonucleotide platforms	Ligand-specific cellular and niche-directed targeting	Cell population determined by the selected targeting ligand	Provides receptor-dependent delivery to selected cell populations	Selective delivery to the intended cell population with reduced off-target exposure	Cell-selective; unvalidated

Candidate interventions and downstream effects in this table are SOIRM hypotheses unless a cell explicitly states established or emerging evidence. They should not be interpreted as demonstrated clinical outcomes.

The table summarizes major oligonucleotide classes and their candidate applications within SOIRM. Proposed effects on malignant and nonmalignant compartments are hypotheses and do not represent demonstrated clinical benefit in MM.

## 2 Evidence base for resistance-supporting bone marrow states

The principal cellular and noncellular components of the resistance-supporting marrow microenvironment are summarized in **Table 2** and **Figure 1**.

**Table 2:** Components of the multiple myeloma bone marrow microenvironment associated with treatment resistance.

Bone marrow component	Biological function	Resistance-supporting role in the bone marrow microenvironment	Dominant spatial resistance niche	SOIRM role in the bone marrow microenvironment	Candidate intervention	Evidence status and proposed scope
**Malignant plasma cells**	Malignant clonal proliferation and monoclonal immunoglobulin production	Adaptive evolutionary engine driving microenvironmental modification	Potential involvement across several proposed states; spatial assignment requires validation	**Evolutionary driver of microenvironment-mediated resistance**	ASOs, siRNAs, multi-target oligonucleotide strategies	Multicompartment effect hypothesized
Mesenchymal stromal cells (MSCs)	Structural support and tissue homeostasis	Coordinate stromal signalling, adhesion, cytokine production, and metabolic support	Retention; Cytokine-Survival	Structural organizer of the local microenvironment	ASOs targeting IL-6/TGF-β, siRNAs	Multicompartment effect hypothesized
Regulatory T cells (Tregs)	Maintenance of physiological immune tolerance	Sustain local immune suppression and inhibit cytotoxic immunity	Immune-Suppression	Immune tolerance stabilizer	CpG ODNs, miRNA therapeutics, anti-TGF-β oligonucleotides	Potentially broad; unvalidated
Myeloid-derived suppressor cells (MDSCs)	Regulation of innate immune responses	Reinforce immune escape and suppress T-cell activation	Immune-Suppression	Innate immune suppressive amplifier	CpG ODNs, anti-miRNA therapeutics	Potentially broad; unvalidated
Tumour-associated macrophages (M2 phenotype)	Tissue remodeling and inflammatory regulation	Promote stromal activation, angiogenesis, and immunosuppressive signaling	Immune-Suppression; Osteoimmune	Inflammatory modulator of the local microenvironment	miRNA therapeutics, immune-regulatory oligonucleotides	Potentially broad; unvalidated
Osteoclasts	Physiological bone remodeling	Generate osteoimmune conditions that reinforce malignant-cell persistence	Osteoimmune	Mediator of bone remodeling and local immune effects	RANKL-directed oligonucleotides, regulatory miRNAs	Potentially broad; unvalidated
Extracellular matrix (ECM)	Tissue architecture and mechanical support	Maintain adhesion-mediated resistance and spatial protection	Retention; MRD-Persistence	Spatial microenvironmental scaffold	Interventions targeting cell-matrix adhesion	Potentially broad; unvalidated
Bone marrow vasculature	Oxygen and nutrient delivery	Supports angiogenesis, metabolic adaptation, and therapeutic shielding	Cytokine-Survival; MRD-Persistence	Source of vascular and metabolic support	Aptamer-guided delivery systems, anti-angiogenic oligonucleotides	Target-specific or limited

Candidate interventions and downstream effects are SOIRM hypotheses unless explicitly identified as established or emerging evidence.

The table summarizes the malignant, stromal, immune, vascular, bone, and extracellular matrix components associated with MM progression, immune escape, treatment resistance, MRD persistence, and relapse. Candidate oligonucleotide interventions are proposed for experimental testing and have not been clinically validated.

Bold text is used for emphasis only and does not indicate statistical significance or a separate level of evidence.

**Figure 1:**
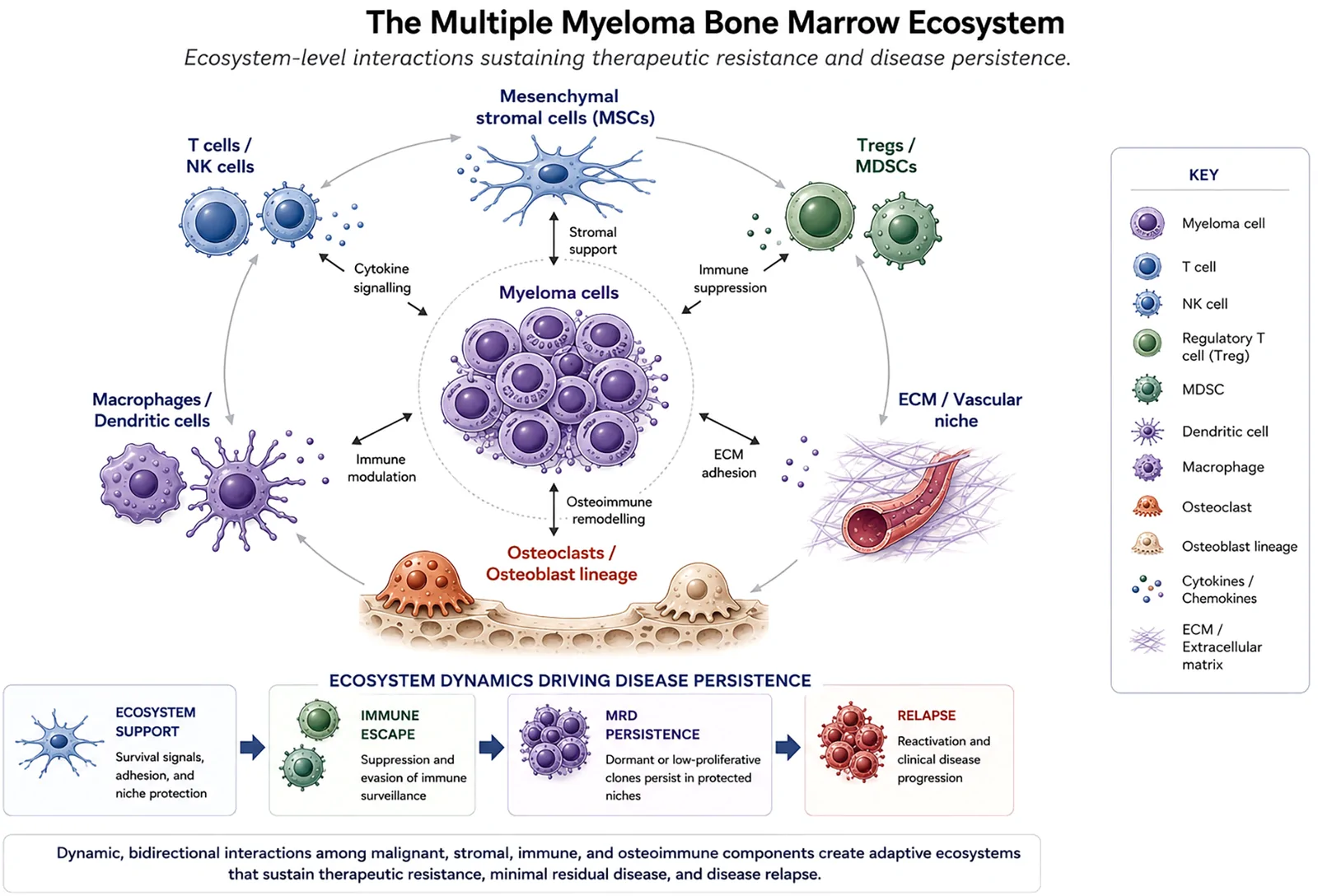
Cellular and molecular components of the multiple myeloma bone marrow microenvironment. The figure shows malignant plasma cells in relation to mesenchymal stromal cells, T-cell subsets, natural killer cells, dendritic cells, macrophages, myeloid-derived suppressor cells, osteoclasts, cytokines, extracellular matrix, and vascular components. Experimental studies support roles for selected interactions in immune suppression, stromal support, bone disease, treatment resistance, residual disease, and relapse. The proposed benefit of disrupting several interactions simultaneously remains an unvalidated SOIRM hypothesis.

### 2.1 Stromal adhesion, inflammatory signaling, and transcriptional plasticity

Established finding. MM cells receive survival and drug-resistance signals through adhesion and cytokine pathways. Disruption of CXCR4 with AMD3100 mobilized myeloma cells from protective interactions within the bone marrow and increased treatment sensitivity in preclinical models (12). Earlier co-culture experiments showed that stromal contact can alter dependence on IL-6/gp130/STAT3 signaling (13). More recent patient-linked work moves beyond isolated pathways: inflammatory stromal populations were identified by single-cell analysis (4), and stromal contact produced reproducible chromatin and transcriptional remodeling in malignant cells, with an adverse program associated with dissemination and outcome (2).

Emerging evidence. The IL-1β-neutrophil-stromal study linked marrow inflammation to BAFF-mediated support using patient samples and mechanistic experiments (5). It provides evidence for a defined multicellular pathway rather than a nonspecific description of microenvironmental communication. Important limitations include the experimental context, patient heterogeneity, and the absence of validated predictive thresholds.

SOIRM hypothesis. A prespecified combination of spatial proximity, ligand-receptor activity, and functional dependence may define a cytokine-supporting state. It should be considered therapeutically relevant only if perturbing the proposed pathway changes malignant-cell fitness and a prespecified nonmalignant-cell readout beyond the effect of direct tumor-cell cytotoxicity.

### 2.2 Immune composition and response to immunotherapy

Established and emerging findings. Relapsed MM exhibits altered immune composition and malignant-cell-mediated immunosuppression (14). Osteoclasts can directly contribute to an immunosuppressive marrow environment (15). In a clinically annotated analysis of BCMA × CD3 bispecific T-cell engager therapy, the pre-existing marrow T-cell landscape and subsequent T-cell state transitions were associated with response, providing direct evidence that treatment efficacy depends in part on the recipient immune context (16). Genetically heterogeneous mouse models have also reproduced immune-evasion mechanisms and differential immunotherapy responses observed in patients (17).

Author interpretation. These studies support measuring the immune microenvironment but do not justify classifying all forms of immune suppression as a single state. T-cell dysfunction, myeloid suppression, antigen loss, stromal inflammation, and osteoclast-mediated effects require distinct assays and may require different interventions. SOIRM therefore treats them as separate candidate states whose boundaries must be defined empirically.

### 2.3 Spatial heterogeneity and focal lesions

Established finding. Multi-region sequencing demonstrated spatial genomic heterogeneity in MM (18). Subsequent single-cell work refined this result: paired osteolytic lesion and marrow samples from 10 patients showed site-dependent transcriptional programs and treatment-associated shifts (6), whereas broader multi-region profiling revealed lesion-specific subclones, spatial transcriptional plasticity, and regional differences in macrophage abundance and therapeutic-target expression (7). Longitudinal sampling further showed that focal lesions can act as reservoirs or evolutionary hotspots for relapse (1).

Emerging evidence. Current spatial studies are relatively small, use different platforms, and sample only a fraction of total skeletal disease. Spatial structure can therefore be clinically important without being uniformly measurable or causal. A proposed resistance niche must not be inferred solely from colocalization; it requires reproducibility, functional perturbation, and association with a prespecified outcome.

### 2.4 MRD: tumor burden versus residual-state biology

Established finding. Standardized next-generation flow is a sensitive method for MM MRD detection (19), and MRD status is strongly associated with survival (20, 21). These methods quantify residual malignant cells but do not directly measure the state of the supporting tissue. Multi-site studies demonstrate that a single-site aspirate can overlook geographically restricted disease, especially in the presence of focal lesions (1, 18). FDG-PET can also be falsely negative in MM with low hexokinase-2 expression (22).

SOIRM hypothesis. A microenvironmental signature of residual disease should add prognostic information to established MRD testing, imaging, cytogenetics, and treatment variables. If spatial or immune measurements do not improve model calibration, discrimination, or clinical utility beyond these comparators, the proposed signature should not be retained as an independent construct.

## 3 Oligonucleotide platforms: what is demonstrated and what remains proposed

The principal oligonucleotide platforms and their delivery constraints are summarized in **Figure 2**.

**Figure 2:**
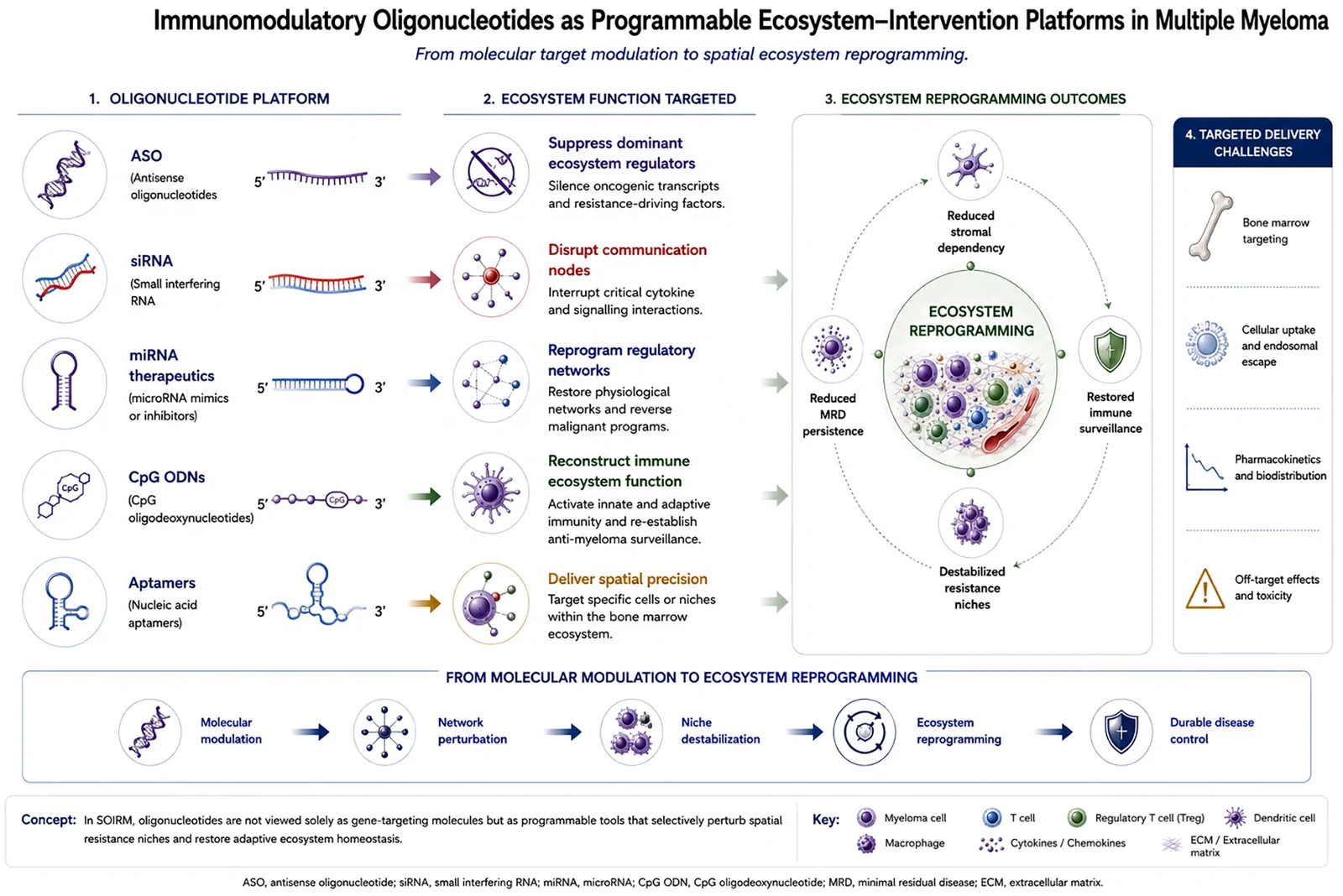
Oligonucleotide platforms relevant to testing SOIRM hypotheses. The figure summarizes antisense oligonucleotides, small interfering RNAs, microRNA therapeutics, CpG oligonucleotides, and aptamer-guided delivery. These platforms differ in mechanism, delivery requirements, toxicity, and the amount of MM-specific evidence. Proposed effects on malignant, stromal, and immune compartments require cell-type-specific delivery, intracellular target engagement, functional assays, and safety assessment. Bulk target knockdown alone is insufficient evidence of a multicompartment effect.

### 3.1 Antisense and siRNA approaches

ASOs can induce RNase H-dependent transcript degradation or modulate pre-mRNA splicing, whereas siRNAs mediate RNA interference (23–25). In oncology, delivery, endosomal escape, exposure heterogeneity, innate immune activation, and on-target toxicity remain major constraints (26–28). These are established modality-level facts, not MM-specific evidence of coordinated effects across malignant and nonmalignant marrow compartments.

Direct evidence in MM is limited. Oblimersen, a BCL2 antisense oligonucleotide, was evaluated in combination with dexamethasone and thalidomide in relapsed MM and later in a randomized study; these trials did not demonstrate sufficient durable clinical benefit to change practice (10, 11). ASO and siRNA platforms can therefore be used to test individual regulators, whereas coordinated effects on malignant, stromal, and immune compartments remain an untested SOIRM hypothesis.

### 3.2 microRNA replacement and inhibition

MicroRNAs are attractive because a single sequence can regulate multiple transcripts, but this breadth also increases the risk of off-target effects and challenges in dose control (29, 30). In MM, synthetic miR-34a reduced tumor growth in preclinical models, and stable nucleic-acid lipid particles improved *in vivo* delivery (8). BCMA aptamer-directed delivery of miR-137 and anti-miR-222 has also been demonstrated in preclinical MM models (9), miR-137, and miR-221/222 in survival, epigenetic regulation, and drug response, but most findings are based on cell-line or xenograft studies.

Author interpretation. Regulation of several transcripts may be useful when redundant pathways maintain resistance. Such an effect should be described as multicompartment modulation only if treatment produces prespecified changes in malignant, stromal, and immune readouts that can be linked to the intended RNA target network.

### 3.3 CpG oligonucleotides and innate immune activation

CpG oligonucleotides activate TLR9-dependent innate and adaptive immune programs (31, 32). Local immunostimulatory oligonucleotide strategies can generate systemic antitumor responses in experimental models outside MM (33). Nanoparticle formulations can enhance CpG-mediated antitumor effects in mouse models (34), while albumin-binding amphiphile conjugates can improve lymph-node targeting of molecular vaccines (35). However, marrow delivery, disease-specific TLR9 biology, and the risk of inflammatory support for myeloma must be evaluated directly. The evidence supports a testable immunologic strategy rather than routine MM application.

### 3.4 Aptamer-guided delivery and spatial selectivity

The clearest MM-specific delivery example is apt69.T, a nuclease-resistant BCMA RNA aptamer that bound to and was internalized by BCMA-positive myeloma cells, delivering miR-137 or anti-miR-222 cargo (9). This is proof of receptor-directed cellular delivery. It does not demonstrate delivery to stromal, myeloid, or spatially defined compartments, and BCMA heterogeneity or antigen loss may restrict its coverage. Within SOIRM, aptamer guidance is therefore an enabling technology whose spatial selectivity must be demonstrated experimentally rather than assumed.

### 3.5 Delivery is the principal translational limitation

For every modality, the relevant denominator is the fraction of target cells in the intended marrow compartment that receive pharmacologically active intracellular oligonucleotides. Plasma stability, renal and hepatic clearance, protein binding, vascular access, extracellular matrix diffusion, cell entry, and endosomal escape determine that fraction (26–28). Bulk-target knockdown can obscure failure in a rare MRD population or in a protected lesion. Future studies should report tissue distribution, cell-type-specific uptake, endosomal release, target engagement, and off-target immune activation, in addition to antitumor effects.

## 4 The SOIRM framework

SOIRM is an author-proposed framework that links three supported observations: MM is spatially heterogeneous, nonmalignant marrow cells can alter drug response, and oligonucleotides can modulate selected molecular targets. Their integration into a spatially targeted treatment strategy has not been established. The Graphical Abstract summarizes the conceptual basis of SOIRM, and **Table 3** distinguishes the supporting evidence from the validation requirements for each proposition.

**Table 3:** Evidence and validation requirements for SOIRM propositions.

SOIRM concept	Current biological evidence	Evidence level	Current support	Critical validation required	Potential experimental approaches	Potential scientific impact
Bone marrow microenvironment contributes to treatment resistance	Established by pathway experiments and recent primary stromal/immune studies (2, 5, 12).	**Established**	Multiple experimental and clinical studies	Validate integrated multicellular causality and clinical relevance	Co-culture or ex vivo marrow models with cell-type-specific perturbation and functional readouts	Provides the established biological basis for SOIRM
Spatial heterogeneity influences myeloma biology	Multi-region and single-cell studies demonstrate spatial genomic, transcriptional, and immune heterogeneity (1, 6, 7).	Established	Strong experimental evidence	Further disease-specific spatial characterization	Spatial transcriptomics, imaging mass cytometry, multiplex IF	Defines the spatial dimension of resistance
Treatment resistance reflects interactions among malignant and nonmalignant cells	Individual mechanisms are established, but integrated multicellular behavior remains incompletely characterized.	Established component mechanisms; integrated claim unproven	Strong indirect evidence	Demonstrate effects that are not explained by isolated cell populations or pathways	Multi-omics integration, systems biology, perturbation studies	Supports microenvironment-focused interpretation of resistance
Spatially defined resistance-associated states can serve as functional units	Spatially defined resistance-associated states are proposed by SOIRM but have not been validated as reproducible therapeutic units.	Hypothesis-Generating	Conceptual integration of existing biology	Demonstrate reproducible spatial organization and biological boundaries	Spatial transcriptomics, digital pathology, spatial proteomics	Establishes the central organizational principle of SOIRM
Proposed six-state classification	Six potentially overlapping states are proposed to organize known mechanisms; a fixed temporal hierarchy has not been established.	Hypothesis-Generating	Based on biological synthesis	Demonstrate dynamic transitions and overlap among niches	Longitudinal spatial profiling, computational trajectory analysis	Provides a testable classification of resistance-associated states
Programmable oligonucleotides may modify several cell compartments	MM preclinical studies support miRNA modulation and BCMA-guided RNA cargo delivery (8, 9); coordinated effects across malignant and nonmalignant compartments remain untested.	Emerging preclinical evidence; integrated claim unproven	Limited MM preclinical evidence for individual targets; coordinated multicompartment effects untested	Demonstrate coordinated microenvironmental modification following intervention	Functional perturbation, organoid models, multi-omics profiling	Provides a testable microenvironment-directed strategy
Microenvironmental modification predicts MRD clearance better than tumor burden alone	Biological rationale exists but prospective evidence is lacking.	Hypothesis	Limited indirect evidence	Correlate microenvironmental modification with MRD dynamics and relapse risk	Longitudinal MRD assessment, spatial immune profiling	Introduces microenvironment-informed MRD monitoring
Microenvironment-informed treatment selection improves disease control	Conceptual framework without prospective clinical validation.	Hypothesis	No direct clinical evidence	Prospective biomarker-guided interventional trials	Adaptive clinical trials, computational modeling of spatial and single-cell data	Defines a hypothesis for prospective clinical testing

The table distinguishes established MM findings, emerging evidence, author interpretation, and SOIRM hypotheses. Each hypothesis is paired with the experiment required to evaluate it.

Bold text is used for emphasis only and does not indicate statistical significance or a separate level of evidence.

The proposed unit of analysis is a functionally validated resistance-associated state rather than a descriptive cell cluster. A candidate state requires: (i) reproducible spatial organization; (ii) a defined cellular and molecular composition; (iii) association with a prespecified treatment phenotype; (iv) functional dependence demonstrated by perturbation; and (v) additional explanatory value beyond tumor genotype, disease burden, and treatment. These criteria prevent the proposed states from being interpreted as established anatomical niches.

The proposed intervention is a prespecified target-delivery pair. The target is selected because perturbation is predicted to alter the candidate resistance mechanism, and the delivery system must reach the relevant cell population at pharmacologically active levels. Success requires prespecified malignant and nonmalignant cell readouts; tumor-cell death alone is insufficient evidence of microenvironmental modification. **Figure 3** illustrates this approach, and **Tables 4** and **5** present testable target-delivery-readout combinations.

**Figure 3:**
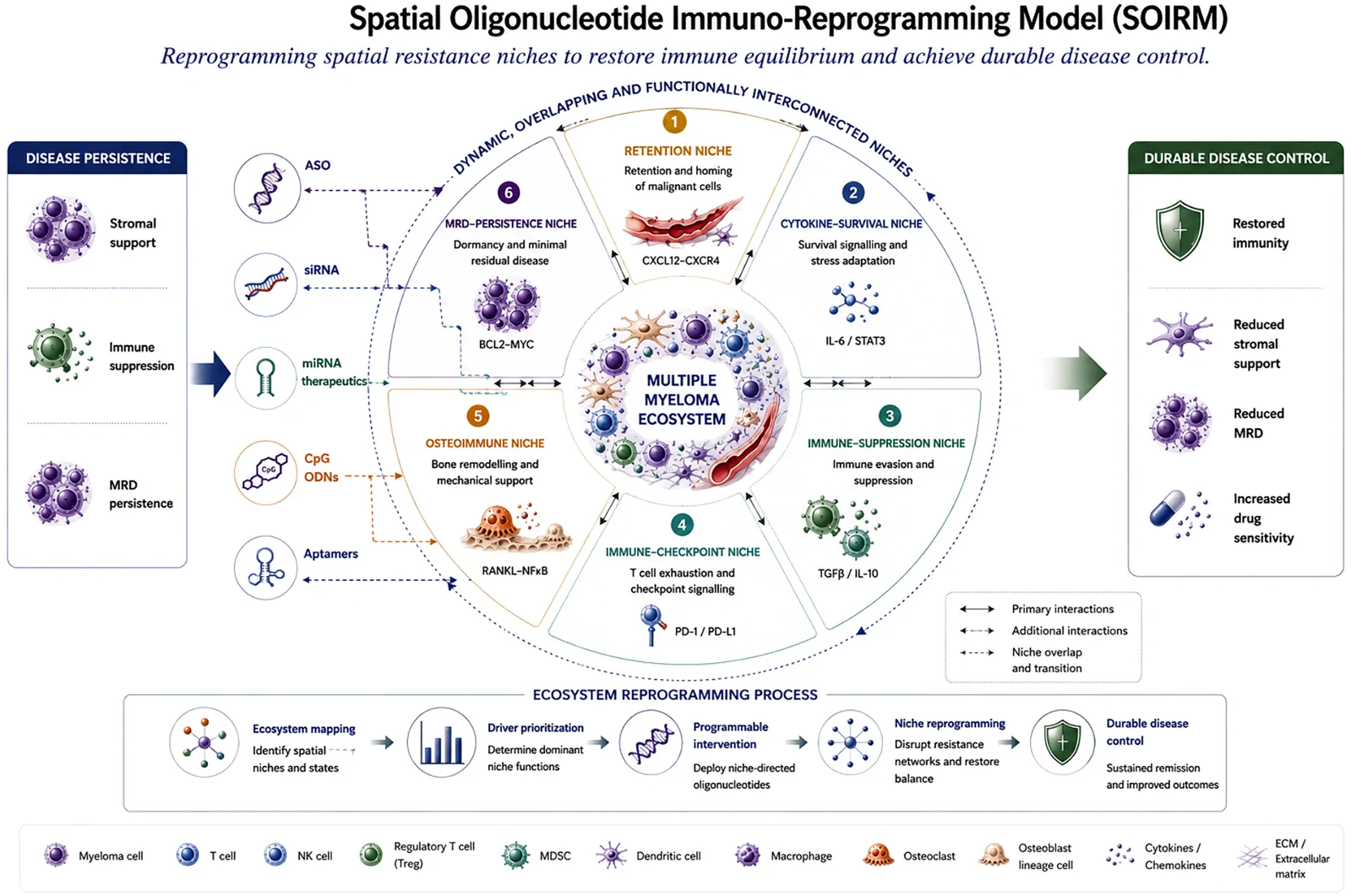
Spatial Oligonucleotide Immuno-Reprogramming Model (SOIRM). The figure links six proposed resistance-associated states—retention, cytokine support, immune suppression, immune-checkpoint signaling, osteoimmune interaction, and MRD persistence—to candidate molecular targets and oligonucleotide strategies. The states and target-delivery combinations are hypotheses. They require reproducible spatial definition, cell-type-specific delivery, functional perturbation, and prospective clinical validation. No pathway shown represents an established MM therapy.

**Table 4:** Candidate spatial resistance-associated states in multiple myeloma.

Proposed functional role	Spatial resistance niche	Dominant biological function	Principal biological drivers	Hypothesized biological consequence	Candidate intervention	Interpretation within SOIRM
Initiation	Retention Niche	Establishes stromal residency and cell-cell and cell-matrix interactions	CXCL12/CXCR4, mesenchymal stromal cells, extracellular matrix	Stable cellular retention and protection from treatment	CXCR4/CXCL12-directed ASOs or siRNAs	Candidate state associated with marrow retention and drug protection
Reinforcement	Cytokine-Survival Niche	Coordinates adaptive multicellular communication	IL-6, JAK/STAT3, IGF-1, BAFF, APRIL	Persistent survival signalling, metabolic adaptation, capacity to sustain resistance	IL-6-, STAT3-, and TGF-β-targeted ASOs; regulatory miRNAs	Candidate state associated with cytokine-mediated survival
Immune Adaptation	Immune-Suppression Niche	Establishes immune tolerance and suppressive microenvironmental organization	TGF-β, IL-10, Tregs, MDSCs, M2 macrophages	Progressive immune escape, reduced cytotoxic surveillance, expansion of permissive immune states	CpG ODNs, anti-TGF-β oligonucleotides, immune-regulatory miRNAs	Candidate state associated with local immune suppression
Functional Stabilization	Immune-Checkpoint Niche	Maintains adaptive immune exhaustion	PD-1/PD-L1 signalling, chronic inflammatory signalling	Durable T-cell dysfunction and sustained immune evasion	PD-L1-directed siRNAs/ASOs, checkpoint-modulating oligonucleotides	Adaptive immune stabilizer preserving capacity to sustain resistance
Structural Adaptation	Osteoimmune Niche	Links skeletal remodeling with changes in the local microenvironment	RANKL, NF-κB, osteoclast activation	Structural remodeling, inflammatory amplification, reinforcement of resistant microenvironmental organization	RANKL-directed oligonucleotides, regulatory miRNAs	Candidate state linking bone remodeling with persistent disease
Persistence	MRD-Persistence Niche	Preserves persistent microenvironmental support following therapy	BCL2, MYC, CXCR4, survival pathways	Maintenance of residual-cell survival networks and microenvironmental support associated with relapse	Multi-target programmable oligonucleotide combinations guided by microenvironmental profiling	Proposed residual-disease state involving several resistance mechanisms

Candidate interventions are hypotheses unless a cell explicitly states established or emerging evidence.

The table describes six states proposed within SOIRM. Their component mechanisms have different levels of support, whereas the six-state classification remains hypothesis-generating and requires spatial, functional, and prospective validation. The row labels describe conceptual functions, not an established temporal sequence; states may overlap and coexist.

**Table 5:** Proposed oligonucleotide strategies and required biological readouts.

Spatial resistance niche	Dominant therapeutic objective	Programmable oligonucleotide strategy	Proposed biological change	Biological readout	Proposed clinical implication (unvalidated)	Testable SOIRM hypothesis
Retention Niche	Disrupt stromal residency and cell-cell and cell-matrix interactions	CXCL12/CXCR4-targeted ASOs or siRNAs	Retention → Mobilization	Reduced stromal adhesion, diminished protective niche occupancy, increased therapeutic accessibility	Enhanced sensitivity to chemotherapy, immunotherapy, and cellular therapies	Disrupting retention may improve exposure to other therapies
Cytokine-Survival Niche	Reduce cytokine-mediated survival signaling	IL-6-, STAT3-, and TGF-β-targeted ASOs; regulatory miRNA therapeutics	Adaptive communication → Network destabilization	Reduced cytokine signalling, impaired metabolic adaptation, decreased capacity to sustain resistance	Potential for deeper response; requires prospective validation	Cytokine signaling may modify treatment response
Immune-Suppression Niche	Restore antitumor immune function	CpG ODNs, anti-TGF-β oligonucleotides, immune-regulatory miRNAs	Immune tolerance → Immune activation	Recovery of antigen presentation, enhanced cytotoxic immunity, reduced suppressive-cell dominance	Improved endogenous and treatment-induced antitumour immunity	Restoration of immune function requires functional confirmation
Immune-Checkpoint Niche	Reverse adaptive immune exhaustion	PD-L1-directed siRNAs/ASOs and checkpoint-modulating oligonucleotides	Immune exhaustion → Functional immune surveillance	Restored T-cell function, improved immune persistence, reduced immune escape	Potential improvement in antitumor immune function; clinical benefit and combination safety unproven	Checkpoint signaling may contribute to persistent T-cell dysfunction
Osteoimmune Niche	Normalize pathological skeletal microenvironmental modification	RANKL-directed oligonucleotides and osteoimmune-regulatory miRNAs	Structural remodeling → Skeletal homeostasis	Reduced osteoclast activation, diminished inflammatory amplification, weakened structural tumour support	Reduced skeletal complications and weaker microenvironmental support	Bone remodeling may support persistent disease
MRD-Persistence Niche	Eliminate persistent microenvironmental support sustaining residual disease	Multi-target programmable oligonucleotide combinations guided by microenvironmental profiling	Persistent state → hypothesized loss of resistance-supporting function	Destabilization of residual survival networks, reduced capacity to sustain resistance, diminished relapse potential	Potential MRD or relapse benefit; not clinically demonstrated	Reducing persistent microenvironmental support may improve disease control

Candidate interventions and downstream effects are unvalidated SOIRM hypotheses unless explicitly stated otherwise.

The table links candidate targets and oligonucleotide platforms to prespecified biological readouts. Proposed multicompartment changes and clinical implications require validation of delivery, target engagement, functional effects, safety, and clinical outcomes.

## 5 Candidate resistance states and evidence boundaries

The six proposed resistance-associated states are illustrated in **Figure 4**.

**Figure 4:**
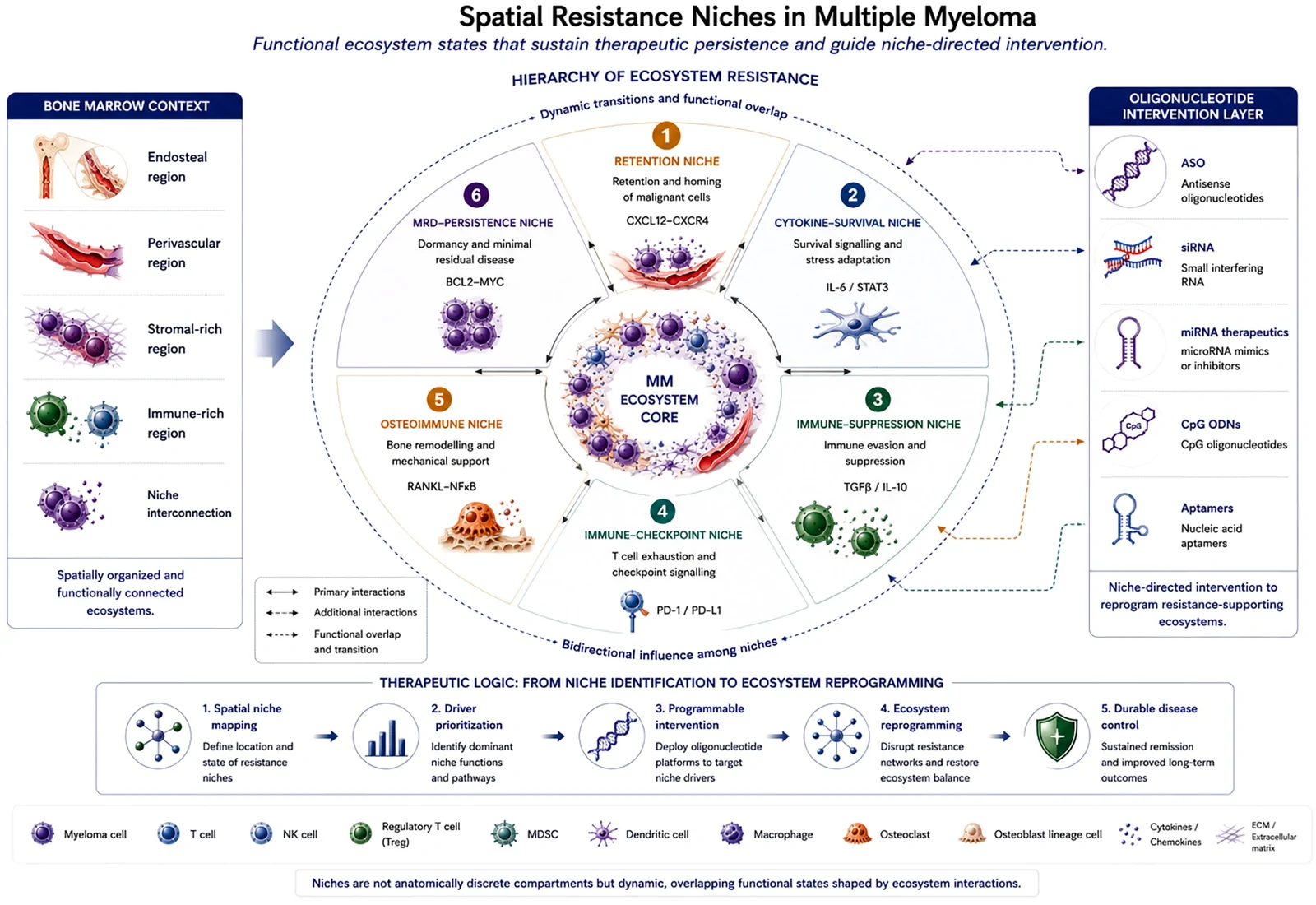
Candidate spatial resistance-associated states in multiple myeloma. The figure maps six candidate states to testable regulators, including CXCL12/CXCR4, IL-6/JAK/STAT3, TGF-β, PD-1/PD-L1, osteoclast-related pathways, and residual-cell survival pathways. The classification integrates established component mechanisms with an author-proposed spatial organization; it does not imply that six reproducible anatomical niches have been validated. Reproducibility, functional dependence, delivery, and additional clinical value must be demonstrated separately for each state.

### 5.1 Retention state

The CXCL12-CXCR4 axis provides direct preclinical evidence of marrow retention and drug protection: pharmacologic disruption of CXCR4 mobilizes MM cells, thereby increasing treatment sensitivity (12). SOIRM interprets this as a candidate retention state. Whether a spatially restricted retention state predicts relapse or benefits from oligonucleotide rather than pharmacologic disruption remains unproven.

### 5.2 Cytokine-survival state

Stromal IL-6/STAT3 signaling and TGF-β-mediated immune regulation contribute to MM survival and treatment context (13, 36). The strongest recent multicellular example is the IL-1β-neutrophil-stromal BAFF axis (5). SOIRM hypothesizes that patients can be stratified by dependence on a defined cytokine circuit; such stratification requires functional assays and cannot be inferred from cytokine abundance alone.

### 5.3 Immune-suppressive and checkpoint states

Immune composition changes with disease and therapy, and pre-existing T-cell states correlate with bispecific-engager response (14, 16). PD-1/PD-L1 biology in MM has not translated into a straightforward benefit from checkpoint blockade (37, 38), illustrating that the presence of this pathway does not equate to therapeutic dependence. SOIRM therefore separates descriptive immune exhaustion from a functionally validated, targetable state.

### 5.4 Osteoimmune state

Osteoclasts promote bone destruction and can suppress antitumor immunity (15). Osteolytic lesions also contain site-specific plasma-cell programs associated with bone disease (6). These findings support an osteoimmune interaction, but the proposed state must be tested against simpler measures such as lesion burden, bone turnover markers, and plasma cell genotype.

### 5.5 MRD-persistence state

MRD positivity is prognostic across studies (20, 21), and next-generation flow provides standardized sensitive detection (19). SOIRM proposes that residual tissue organization may distinguish patients with similar measured tumor burden. The hypothesis requires paired baseline/remission biopsies, imaging, standardized MRD, and longitudinal relapse endpoints; it is currently unsupported by prospective clinical evidence.

## 6 Experimental and translational validation roadmap

**Figure 5** provides a conceptual overview of the proposed translational pathway; the validation stages below specify the tests required before clinical application.

**Figure 5:**
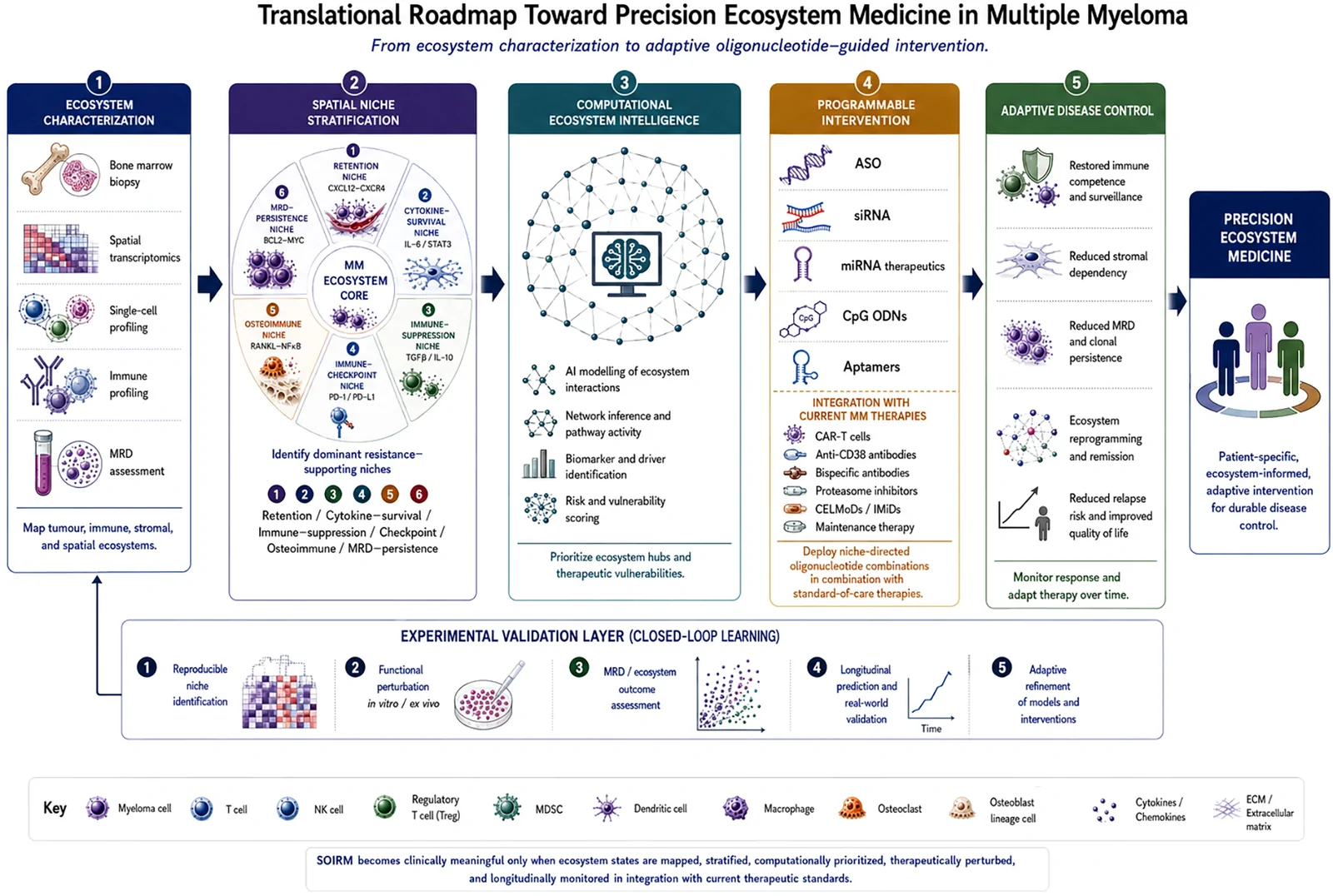
Experimental and translational validation pathway for SOIRM. The figure presents spatial profiling, single-cell analysis, computational integration, delivery studies, pharmacologic validation, and longitudinal assessment as research stages. Patient-specific use and clinical benefit remain hypotheses that require reproducible definitions of the state, adequate tissue delivery, safety assessment, external validation, and prospective clinical testing.

Stage 1—discovery. Prospectively collect spatially preserved marrow trephines, aspirates, imaging-defined focal-lesion samples when clinically justified, and matched peripheral blood. Spatial transcriptomic or proteomic data can be integrated with single-cell reference profiles to identify candidate states. Studies should include technical replicates, negative controls, and predefined criteria for spatial enrichment rather than relying on *post hoc* visual interpretation.

Stage 2—functional validation. Reconstruct candidate states in co-culture, organoid, ex vivo marrow, or genetically heterogeneous mouse models. Perturb one regulator at a time and quantify tumor viability, stromal state, immune function, cytokine output, and spatial organization. The preclinical models described by Larrayoz et al. illustrate how to link genetically heterogeneous MM models to clinically relevant immunotherapy responses (17).

Stage 3—delivery validation. Compare naked and formulated oligonucleotides with respect to pharmacokinetics, biodistribution, cell-type-specific uptake, endosomal escape, target knockdown, and toxicity. A BCMA aptamer-cargo construct can test malignant-cell selectivity (9), whereas non-tumor compartments will require alternative ligands or nanoparticles. Delivery failure must be distinguished from target failure.

Stage 4—incremental clinical validation. Test whether a candidate state predicts treatment response, MRD persistence, or relapse beyond the effects of established covariates. Models should undergo external validation and calibration, with clinical utility assessed by interpretable decision-analytic methods. The biomarker should first be evaluated prospectively without influencing treatment before it is used for treatment selection.

Stage 5—interventional testing. Early trials should enroll a biomarker-defined population, use a prespecified target-delivery pair, confirm pharmacodynamic activity in the intended compartment, and include standard safety endpoints. A positive study should demonstrate target engagement, the predicted changes in malignant and nonmalignant compartments, and a clinically meaningful outcome.

## 7 Integration with current MM therapy

SOIRM is intended to complement rather than replace tumor-directed therapy. Proteasome inhibitors, immunomodulatory drugs, anti-CD38 antibodies, T-cell engagers, and CAR T cells produce distinct selective pressures and immune effects. Any SOIRM-based combination should therefore be treatment-specific. For example, an experimentally validated inflammatory stromal state could be tested as a modifier of response to T-cell engagers. In contrast, a retention-associated state could be evaluated for effects on drug exposure or cellular-therapy trafficking. Interaction studies are essential because immune stimulation, cytokine modulation, and disruption of marrow retention may also increase toxicity.

The framework does not support empirical combinations of multiple oligonucleotides. Each additional component increases manufacturing complexity, uncertainty about tissue exposure, innate immune effects, and the difficulty of attributing benefit or toxicity. Combination development should proceed from validated single-target perturbations, formal synergy testing, and dose-response studies.

## 8 Limitations and risks

The central limitation is the absence of direct evidence that the proposed six states can be reproducibly distinguished, functionally validated, and used to guide treatment. Current spatial MM cohorts are small, sampling is anatomically incomplete, and platforms differ in resolution and analyte coverage. Bone marrow aspirates distort spatial relationships, whereas trephines introduce decalcification and RNA-quality constraints. Temporal evolution further limits any static classification.

The oligonucleotide evidence is also substantially less mature than the spatial and microenvironment evidence. Most MM studies demonstrate target modulation or xenograft activity, not the remodeling of patient marrow. Delivery to rare residual cells and non-malignant compartments, off-target gene regulation, innate immune activation, marrow toxicity, and heterogeneous target expression are unresolved. The negative or inconclusive clinical history of some ASO strategies, including oblimersen, argues against extrapolating from molecular plausibility to efficacy (10, 11).

A conceptual risk is retrospective relabeling of any resistance mechanism as an SOIRM state. The operational criteria in Section 4 are intended to prevent this. The framework should be narrowed if state boundaries are not reproducible, abandoned if spatial information does not add predictive value, and modified if oligonucleotide delivery cannot safely reach the relevant cell populations.

## 9 Conclusion

Primary MM studies demonstrate spatially divergent subclones, inflammatory stromal programs, multicellular cytokine pathways, and immune states associated with treatment response. These findings support evaluation of tissue context alongside tumor genotype, but they do not establish microenvironment-directed oligonucleotide treatment. Direct MM evidence shows preclinical modulation of selected RNAs and BCMA-guided RNA delivery, but it remains insufficient to support clinical intervention targeting spatially defined resistance states.

SOIRM should therefore be regarded as an experimental framework. Its critical tests are whether a reproducible spatial resistance-associated state can be identified, selectively perturbed, and shown to add predictive or therapeutic value beyond current models. Failure at any stage should lead to revision or rejection of the framework. This makes SOIRM a falsifiable research program rather than an established therapeutic model.

## Data Availability

The datasets presented in this study can be found in online repositories. The names of the repository/repositories and accession number(s) can be found in the article/**Supplementary Material**. Further inquiries can be directed to the corresponding author.
